# Reducing Health Inequities Through Total Knee Arthroplasty: An Experience From Bhutan

**DOI:** 10.1002/hsr2.70459

**Published:** 2025-02-10

**Authors:** Nomina Pradhan, Monu Tamang, Sagar Rai, Choeda Gyaltshen, Choney Dema, Mimi Lhamu Mynak, Michael Canestrari, Kenneth Christopher Sands, Kuenzang Wangdi

**Affiliations:** ^1^ Department of Orthopaedic Surgery Central Regional Referral Hospital Gelephu Bhutan; ^2^ Department of Physiotherapy Central Regional Referral Hospital Gelephu Bhutan; ^3^ Central Regional Referral Hospital Gelephu Bhutan; ^4^ National Medical Services Ministry of Health Thimphu Bhutan; ^5^ Home Health Care of Florida Melbourne Florida USA; ^6^ Melbourne Regional Medical Center Melbourne Florida USA

**Keywords:** health equities, osteoarthritis, surgical camp, total knee arthroplasty

## Abstract

**Background and Aims:**

Bhutan is a low–middle‐income country with a 0.7 million population with a high burden of musculoskeletal conditions. Recognizing the high burden of osteoarthritis, total knee arthroplasty (TKA) was launched in the country in 2022. However, Bhutan continues to refer complicated cases to India. In 2024, International Operation, a US‐based nonprofit secular and humanitarian organization, conducted a TKA camp in Bhutan. This perspective aims to report about the camp and discuss how such camps help reduce healthcare disparities.

**Method:**

We compiled data on patients who underwent total knee or hip arthroplasty in last 7 years from the registry maintained at National Referral Hospital of Bhutan. We shared our experience of hosting TKA camp and discuss how such camps might help reduce healthcare disparities.

**Result:**

In last 7 years, Bhutan referred increasing number of patients for total knee and hip arthroplasty to India. Royal Government of Bhutan spends Nu. 250,000 (approximately US$3000) per patient excluding expenses for travel, logistics, and medications. A team from International Operation conducted TKA on 31 patients during the camp.

**Conclusion:**

Such camps would help reduce the healthcare disparities in low‐ and middle‐income countries.

## 1 Introduction

Health equity, in essence, is the absence of systematic disparities in healthcare among groups of people with different races, ethnicities, religious groups, and socioeconomic status, including wealth, power, or prestige [1]. Health equity is a pivotal component of social justice in health and human development [1]. The WHO's Commission on Social Determinants of Health in 2008 identified factors influencing the health equity in policies, programs, and services, and they include socioeconomic conditions, health finance, human resources, daily living conditions, sociopolitical systems, and overall governance of health systems [2]. The commission calls for the attention to reduce health disparities to achieve one's right to health—a state of complete physical, mental, and social well‐being and not merely the absence of disease or infirmity. Likewise, the health disparities vary from one country to another, from rural to urban areas in the country.

Bhutan is a lower‐middle‐income country located between India and China in the eastern Himalayas, with a population of 0.7 million. The country is reporting an increasing incidence of musculoskeletal disorders, including osteoarthritis, which was the fourth most common morbidity leading to hospital visits in 2023 [3]. Like many low‐ and middle‐income countries [4], Bhutan does not have comprehensive data on osteoarthritis, leading to underreporting of disease burden, disability, compromised quality of life, and financial implications. The overall burden of osteoarthritis is increasing globally, with a disproportionate increase in low‐ and middle‐income countries, reflecting epidemiological and sociodemographic shifts [4]. Osteoarthritis commonly involves the knee, hip, and hand joints, with knee osteoarthritis being the most common form of osteoarthritis [4].

Addressing the growing burden of osteoarthritis is associated with Sustainable Development Goal (SDG) 3 in ensuring healthy lives and promoting well‐being for all at all ages and SDG 3.8 in achieving universal health coverage. However, healthcare systems of low‐ and middle‐income countries are overwhelmed in tackling infectious diseases, the emerging burden of noncommunicable diseases, and life‐threatening conditions, including maternal, nutritional, and child health. Musculoskeletal disorders, on the other hand, receive little attention because they are rarely fatal and are often considered irreversible conditions [4], increasing the health disparities among different disease conditions. In this perspective, we report on the management of osteoarthritis in Bhutan, the first total knee arthroplasty (TKA) camp in the country, and discuss how such initiatives can reduce health inequities in low‐ and middle‐income countries.

## 2 Healthcare System of Bhutan

As mandated by its constitution, Bhutan provides all levels of allopathic and traditional healthcare services free of cost. As of 2023, Bhutan's three‐tier healthcare system had 187 primary healthcare centers and 50 subposts at the grassroots level, 51 hospitals at the secondary level, and three tertiary hospitals [3]. With the Health Transformation in 2023, all clinical services are looked after by National Medical Services under the Ministry of Health. Although National Traditional Medicine Hospital is located separately in Thimphu, all traditional medicine units across the country are colocated with the allopathic hospitals [3]. Bhutanese Traditional Medicine is based on the Buddhist philosophy of imbalance in five elements, three humors, and secretions of the human body, resulting in illness [5]. The treatment consists of behavioral modifications, the use of herbal medicine, both invasive and noninvasive therapies, and spiritual healing [5].

Patients with musculoskeletal disorders, including osteoarthritis, can present at any level of hospital. However, the health‐seeking behaviors of musculoskeletal patients in Bhutan are quite complex. Patients first try home remedies such as herbal remedies and massage, and literate patients tend to browse the Internet for exercises and home remedies [6]. Musculoskeletal pain patients in rural communities seek help and intervention from local healers. The local healing system includes informal traditional knowledge‐based therapies handed down through generations by cultural learning, apprenticeship with the master healer, or other traditional means of knowledge transmission. People visit hospitals—both allopathic and traditional medicine—often when home remedies and local healing systems fail, or vice versa [6]. Typical management of musculoskeletal pain disorders in hospitals consists of radiological imaging to rule out sinister red flags, prescriptions of pain medication, and physiotherapy management [6].

As of 2024, the country had 16 orthopedic surgeons providing specialized services in six hospitals. Diagnostic services such as X‐rays are available in all hospitals. Though Total Hip Replacement was referred to as the “operation of the century” for its effectiveness in the treatment of severe osteoarthritis [7], the Royal Government of Bhutan referred all severe osteoarthritis patients requiring replacement surgery to India until 2022 [8]. The first Total Knee Replacement surgery in the country was performed on December 30, 2022. Following the launch of knee replacement surgeries in Bhutan, lots of patients registered for the surgeries, resulting in long waiting times due to limited resources and unavailability of adequate operating rooms. Meanwhile, Bhutan continues to refer complicated cases to India for surgery. Therefore, the Total Knee Replacement Camp by Operation International not only reduced the waiting time for the patients but also provided care for complicated cases. Operation International, a US‐based nonpolitical, secular, and humanitarian organization, conducted the first Total Knee Replacement Camp in the country at Gelephu in 2024.

## 3 Burden of Osteoarthritis in Bhutan

Bhutan lacks comprehensive data on osteoarthritis to evaluate the financial burden, impact on quality of life, and disability. However, the National Referral Hospital alone recorded more than 100 patients in a year with severe osteoarthritis who could benefit from replacement surgery [8]. Due to a lack of joint arthroplasty surgeons in the country, patients with severe osteoarthritis were referred to India for arthroplasty (Table 1). There was a significant reduction in referrals to India in 2020–2021 owing to the COVID‐19 pandemic and associated travel restrictions. A panel of specialists at Jigme Dorji National Referral Hospital decides the patient referrals to India and the Royal Government of Bhutan spends about Nu. 250,000 (approximately US$3000) per patient for Total Knee Replacement alone, excluding the expenses for medications, travel, and logistics [8]. Since the launch of joint arthroplasty in Bhutan, as of June 10, 2024, a total of 34 cases were operated. However, complicated and revision cases are still referred to India. Currently, Bhutan has two arthroplasty surgeons.

**Table 1:** Summary of patients who underwent total hip and knee replacement, Bhutan, 2017–2024.

Years	THA^a^	TKA^b^	Total	Male	Female	Place
2017–2018	5	30	35	17	18	India
2018–2019	8	42	50	25	25	India
2019–2020	8	33	41	15	26	India
2020–2021	1	1	2	2	0	India
2021–2022	18	23	41	20	31	India
2022–2023	23	31	54	21	33	India
2023–2024	19	24	43	24	19	India
2022–2024	0	34	34	17	17	Bhutan (JDWNRH)
2024	0	31	31	10	21	Bhutan (Operation International TKA camp at CRRH)

Abbreviation: CRRH, Central Regional Referral Hospital.

a THA = total hip arthroplasty.

b TKA = total knee arthroplasty.

## 4 TKA Camp in Bhutan

The initial discussions for conducting a TKA camp in Bhutan began in 2020. The idea was proposed by Operation Walk, a volunteer medical humanitarian organization that provides the gift of mobility through life‐changing joint replacement surgeries at no cost for those in need in the United States and globally. Recognizing the potential benefits of the TKA camp, the Ministry of Health and relevant stakeholders engaged in detailed planning to facilitate the camp. Subsequently, a team member from Operation Walk collaborated with Operation International to enhance the resources and expertise available for the camp. This collaboration aimed to leverage the strengths of both organizations to ensure a successful and impactful camp.

In early 2023, Operation International wrote to the Ministry of Health, Royal Government of Bhutan about the camp. The National Medical Services contacted three referral hospitals to seek their support. Among them, the Medical Superintendent of Central Regional Referral Hospital (CRRH) in Gelephu, expressed keen interest in hosting the camp. CRRH is located in southern Bhutan, near the international border with India, and serves as one of the three tertiary hospitals in the country. The Medical Superintendent consulted the orthopedic surgeon of the hospital to ensure that the idea was evaluated from a clinical perspective. After collaborative discussions with the National Medical Services, the hospital agreed to host the specialized surgery camp with the primary objective of enhancing the quality of life and functional independence of people with severe knee osteoarthritis. The hospital appointed an orthopedic surgeon as team leader to organize the camp at the hospital. Further discussions between the hospital and Operation International began as early as August 2023 and scheduled a week‐long camp in April 2024.

The orthopedic team of the hospital screened potential patients for surgery and provided information to the Operation International team for obtaining implants of specific sizes and certain specifications. The implants were donated by the Enovis and Meril companies. The overall coordination for the camp was facilitated by National Medical Services in collaboration with the hospital. Logistic arrangements, including visa works, route permits, import authorization of medical supplies, and temporary practice licenses, were arranged by National Medical Services. Meanwhile, the hospital team announced the camp through their official Facebook page on March 11, 2024. A total of 1046 patients with knee conditions visited the orthopedic OPD of the hospital from March 11 to March 30, 2024. However, not all patients were willing to undergo surgery for various reasons, such as cultural and superstitious beliefs, fear of undergoing surgery, preference for nonsurgical treatments, and misconceptions about the treatment process. Therefore, only 74 patients provided their consent for the surgery.

The hospital team leader conducted several rounds of internal coordination meetings between the unit heads and unit in‐charges of the hospital. Discussions ranged from logistical arrangements, such as the availability of beds in the Surgery Ward, to pooling healthcare professionals from different wards and hospitals. The hospital also mobilized backup instruments and medications from other hospitals. Additionally, surgeons of different specialties either preponed or postponed elective surgeries to accommodate patients undergoing Total Knee Replacement in the Surgery Ward. Of the three Operating Theaters, two were assigned to the TKA camp, leaving one Operating Theater for emergency cases. This strategic planning helped to optimize the workflow, prioritize patient care, and ensure that the camp operated effectively with the available resources without hampering the routine and emergency services of the hospital.

The second phase of screening was conducted from April 16 to April 24, 2024. Out of 74 patients, 49 were selected for surgery. Clinical criteria for patient selection were debilitating persistent pain, failed conservative management, severe osteoarthritis confirmed by radiographs, and knee deformity [9]. The selected patients underwent comprehensive screening, including clinical assessment, radiological imaging, laboratory evaluations, preanesthesia check‐up, electrocardiograms, and functional assessments. All selected patients underwent preoperative rehabilitation.

The 21‐membered Operation International team arrived at Paro on April 27 and traveled directly to Gelephu via road. The team consisted of surgeons, anesthetists, nurses, and physiotherapists. The National Medical Services arranged all the logistics, including bus services, to travel from hotel to hospital and vice versa. The Bhutanese team, which consisted of two arthroplasty surgeons, two orthopedic surgeons, one anaesthesiologist, nurse anesthetists, nurses, and physiotherapists, was equally involved in the camp.

Coinciding with the astrologically auspicious day on April 28, the Hospital Lama (monk) conducted purifying rituals for the success of the camp. After the rituals, two orthopedic surgeons of the hospital presented details of selected patients to the International Operation team. Following the presentation, the surgeons and doctors met the patients in person for further clinical evaluation. Out of 49 patients, surgery for 18 patients was deferred due to their underlying medical conditions, such as uncontrolled hypertension, diabetes, asthma, and acute upper respiratory tract infection, to avoid postoperative complications. After a thorough evaluation, three surgeries were performed on the first day.

The patients were admitted to the ward 1 day before their surgery. Intensive postoperative rehabilitation was initiated immediately to harvest optimum clinical and functional outcomes [10]. The majority of patients were discharged on the second postoperative day. They were educated about potential complications and prophylactic measures and prescribed adequate pain medications and home exercise programs. Out of the 31 patients, 21 were female; three patients underwent bilateral knee replacement. The eldest patient was an 87‐year‐old lady, and the youngest was a 45‐year‐old male.

## 5 Bridging Health Inequities Through Surgery Camps

### 5.1 Access to World‐Class Specialized Care

Many low‐ and middle‐income countries face significant barriers to accessing specialized surgical care such as TKA due to limited financial resources, inadequate healthcare infrastructure, and a shortage of human resources [11]. Such factors deprive patients of specialized surgeries resulting in increased health disparities [11]. Furthermore, some surgeries like TKA are not prioritized because it is not a life‐saving surgery. Therefore, surgery camps provide an opportunity for patients to avail of specialized services from skilled and experienced human resources in the field.

### 5.2 Improved Quality of Life and Functional Independence

THA is lauded as the “operation of the century” for its effectiveness to alleviate pain, improve the quality of life, and functional independence [7]. Enhancing physical functions enables patients to participate in both social and recreational activities, fulfilling the WHO's definition of health. For example, older adults in Bhutan value religious activities, including stupa circumambulation and attending religious gatherings, which enhance their mental well‐being. Furthermore, the restoration of functional independence facilitates engagement in economically productive activities, which is particularly beneficial for working‐age individuals whose livelihoods may have been compromised by osteoarthritis. Overall, TKA camp benefits the individual and society through improved productivity and reduced healthcare costs associated with long‐term disability and pain management.

### 5.3 Reduced Financial Burden

Surgery camps in low‐ and middle‐income countries significantly reduce the financial burden on the healthcare system and out‐of‐pocket expenditure. For example, the Royal Government of Bhutan spends Nu. 250,000 (approximately US$3000) per patient referred to India for TKA [8], excluding medication costs, travel and logistics, and out‐of‐pocket expenditures. However, the camp provided high‐quality care at little to no cost, eliminating substantial health expenditure.

### 5.4 Knowledge and Skills Development

Such a camp also facilitates capacity‐building and knowledge transfer to local health human resources. By working alongside local health human resources, visiting surgeons and healthcare professionals provide hands‐on training to build the capacity of local surgeons and healthcare professionals. Moreover, these partnerships often lead to the donation of advanced medical equipment and supplies, further bolstering the capabilities of local healthcare facilities. Such collaborations are pivotal to reduce the health disparities between high‐ and low‐income countries.

### 5.5 Influence to Provide Specialized Care in the Local Setting

Specialized surgery camps often serve as catalysts for broader programmatic growth of specialized services in the host countries. The success and positive clinical outcomes of such camps generate increased interest and investment in the healthcare sector from various stakeholders, including government bodies, nongovernmental organizations, and private entities within and outside the country. As such, the overall quality of healthcare services in the host country is elevated, leading to better health outcomes for the population.

## 6 Conclusion

Recognizing the huge burden of osteoarthritis, the TKA camp was conducted in Bhutan. Such camps help reduce the health inequities between high‐ and low‐income countries by providing access to world‐class specialized care, improved quality of life and functional independence of patients, reduced out‐of‐pocket expenditure and financial burden on the healthcare system, skills development of local healthcare professionals, and influence to provide specialized care in local settings.

## Author Contributions

**Nomina Pradhan:** conceptualization, methodology, validation, supervision, visualization, project administration, writing – original draft, writing – review and editing. **Monu Tamang:** conceptualization, methodology, writing – original draft, writing – review and editing, resources. **Sagar Rai:** writing – review and editing. **Choeda Gyaltshen:** writing – review and editing. **Choney Dema:** writing – review and editing. **Mimi Lhamu Mynak:** writing – review and editing. **Michael Canestrari:** writing – review and editing. **Kenneth Christopher Sands:** writing – review and editing. **Kuenzang Wangdi:** writing – review and editing, resources, validation.

## Ethics Statement

The authors have nothing to report.

## Conflicts of Interest

The authors declare no conflicts of interest.

### 1 Transparency Statement

Dr Nomina Pradhan affirms that this manuscript is an honest, accurate, and transparent account of the study being reported; that no important aspects of the study have been omitted; and that any discrepancies from the study as planned (and, if relevant, registered) have been explained.

## Data Availability

The data that support the findings of this study are available from the corresponding author upon reasonable request.
